# Worcester Infirmary and the Jubilee Fund

**Published:** 1913-10-04

**Authors:** 


### WORCESTER INFIRMARY AND THE JUBILEE FUND.

At the monthly meeting of the executive committee of Worcester General Infirmary there was some discussion on the Queen's Jubilee Fund (Worcestershire). In reply to a communication which had been made by the infirmary committee to the Trustees of the Fund, the Secretary to the Trustees stated that as yet no allocation of the money had been made among the hospitals in the county. Alderman Leicester reminded the committee that the Jubilee was in 1887, and said he should like to know the real position of the infirmary. It might be that they had no real right to the money, but if they had a vested right they should take means to get it. The Chairman said he understood a certain amount was ear-marked for the infirmary. Mr. Harris moved that Canon Longhurst and Alderman Leicester be asked to make inquiries, which was carried.

